# The Impact of Urinary Catheterization on the Antibiotic Susceptibility of ESBL-Producing Enterobacterales: A Challenging Duo

**DOI:** 10.3390/antibiotics13050462

**Published:** 2024-05-17

**Authors:** Ionela-Larisa Miftode, Andrei Vâță, Radu-Ștefan Miftode, Tudorița Parângă, Mihaela Cătălina Luca, Carmen Manciuc, Amalia Stefana Țimpău, Viorel Radu, Manuel Florin Roșu, Lidia Oana Stămăteanu, Daniela Leca, Dana Teodora Anton-Păduraru, Egidia Gabriela Miftode

**Affiliations:** 1 Department of Infectious Diseases (Internal Medicine II), Faculty of Medicine, University of Medicine and Pharmacy “Gr. T. Popa”, 700115 Iasi, Romania; ionela-larisa.miftode@umfiasi.ro (I.-L.M.); andrei.vata@umfiasi.ro (A.V.); tudorita.paranga@umfiasi.ro (T.P.); mihaela.luca@umfiasi.ro (M.C.L.); doina.manciuc@umfiasi.ro (C.M.); lidia-oana.stamateanu@umfiasi.ro (L.O.S.); daniela.leca@umfiasi.ro (D.L.); egidia.miftode@umfiasi.ro (E.G.M.); 2 St. Parascheva Clinical Hospital of Infectious Diseases, 700116 Iasi, Romania; florin.rosu@umfiasi.ro; 3 Department of Internal Medicine I (Cardiology), Faculty of Medicine, University of Medicine and Pharmacy “Gr. T. Popa”, 700115 Iasi, Romania; amalia.timpau@umfiasi.ro; 4 Department of Urology, Faculty of Medicine, University of Medicine and Pharmacy “Gr. T. Popa”, 700115 Iasi, Romania; 5 Department of Intensive Care Unit, Infectious Diseases Clinical Hospital, 700115 Iasi, Romania; 6 Department of Mother and Child Medicine, Faculty of Medicine, University of Medicine and Pharmacy “Gr. T. Popa”, 700115 Iasi, Romania; dana.anton@umfiasi.ro

**Keywords:** antimicrobial resistance, urinary catheterization, Enterobacterales

## Abstract

Introduction: Antimicrobial resistance (AMR) is currently a growing concern among healthcare providers, underscoring the importance of describing the regional susceptibility profile for common microorganisms that are associated with urinary tract infections (UTIs). This knowledge serves as the foundation for proper empirical therapeutic recommendations tailored to local susceptibility patterns. Results: We found a high prevalence of ESBL-producing strains (36.9%), with *Escherichia coli* and *Klebsiella* spp. being the most prevalent isolated bacteria. Among the catheterized patients, *Klebsiella* spp. emerged as the primary etiology, with a significant correlation between catheterization and *Proteus* spp. (*p* = 0.02) and *Providencia stuartii* (*p* < 0.0001). We observed significant correlations between urinary catheterization and older age (68.9 ± 13.7 years vs. 64.2 ± 18.1 years in non-catheterized patients, *p* = 0.026) and with the presence of an isolate with extensive drug resistance (*p* < 0.0001) or even pandrug resistance (*p* < 0.0001). Susceptibility rates significantly decreased for almost all the tested antibiotics during the study period. Notably, susceptibility was markedly lower among catheterized patients, with the most pronounced differences observed for carbapenems (59.6% versus 83.4%, *p* < 0.0001) and aminoglycosides (37.1% versus 46.9%, *p* = 0.0001). Materials and Methods: We conducted a retrospective study analyzing the susceptibility profiles of 724 extended-spectrum beta-lactamases (ESBL)-producing Enterobacterales isolated from urine cultures. Our focus was on highlighting susceptibility profiles among isolates associated with urinary catheterization and assessing the shifts in the susceptibility rates over time. Conclusions: The constant rise in AMR rates among Enterobacterales presents significant challenges in treating severe infections, particularly among urinary catheterized patients. This trend leaves clinicians with limited or no effective treatment options. Consequently, the development and implementation of personalized treatment protocols are imperative to ensure efficient empirical therapies.

## 1. Introduction

Infections caused by Gram-negative bacteria continue to impose a significant burden on the public health system, profoundly affecting patients’ prognosis [1]. The alarming rise in the production and widespread dissemination of extended-spectrum beta-lactamases (ESBLs) represent the foremost mechanism of beta-lactam resistance. ESBLs, typically encoded on plasmids, confer resistance to a broad spectrum of antibiotics, including most penicillins, cephalosporins, and aztreonam, through their enzymatic hydrolysis [2]. Additionally, while ESBLs do not directly deactivate non-beta-lactam agents, microorganisms carrying ESBLs often harbor genes that confer resistance to other antibiotics such as ciprofloxacin, gentamicin, or trimethoprim–sulfamethoxazole [3,4].

Catheter-associated urinary tract infections (CAUTIs) caused by Gram-negative bacilli, particularly from the Enterobacterales family, are of paramount importance, due to the high antimicrobial resistance (AMR) rates associated with these microorganisms [5,6]. This aspect is noteworthy in Eastern Europe, particularly in Romania, where the AMR rates are among the highest in Europe. A report published in 2022 by the Institute for Health Metrics and Evaluation from the Global Research on Antimicrobial Resistance (GRAM) project states that, in Romania, in 2019, there were 16,500 deaths associated with AMR, and 4300 were directly attributable to AMR [7].

The leading pathogen incriminated on this issue is Escherichia coli, followed by Staphylococcus aureus; Klebsiella pneumoniae; Streptococcus pneumoniae; and non-fermenters, such as Acinetobacter baumanii and Pseudomonas aeruginosa. It is important to note that antibiotic resistance is the third leading cause of mortality in Romania, after cardiovascular diseases and neoplasms; remarkably, it surpasses the mortality rates of other prevalent conditions such as stroke, liver cirrhosis, or chronic kidney disease. This highlights the severity of the issue and the need for urgent action to combat it [8].

AMR dynamic variability rates among Enterobacterales strains across different geographical regions, or even among hospitals within the same region, result in resistance data that possess low spatial and temporal reproducibility [9,10]. Consequently, it is more prudent to concentrate concerns regarding the evolution of AMR levels on regional trends rather than fixating on recorded absolute values. Acquiring such data can serve as the foundation for crafting therapeutic recommendations customized to the local susceptibility profile. This approach supplants the reliance on personal experience and international guidelines as primary working tools in clinical practice [11].

Therefore, the objective of this study was to evaluate the incidence of CAUTIs caused by ESBL-producing Enterobacterales among inpatients from northeastern Romania. Additionally, we aimed to delineate the AMR trends of these isolates and discern differences in the susceptibility profiles of isolates associated with urinary catheterization compared to those without. By conducting this assessment, we sought to contribute to the understanding of the epidemiology and antimicrobial resistance patterns of CAUTIs in our region. This knowledge could inform strategies for infection prevention and control, as well as guide empirical antibiotic therapy decisions, ultimately improving patient care and outcomes.

## 2. Results

**Figure 1:**
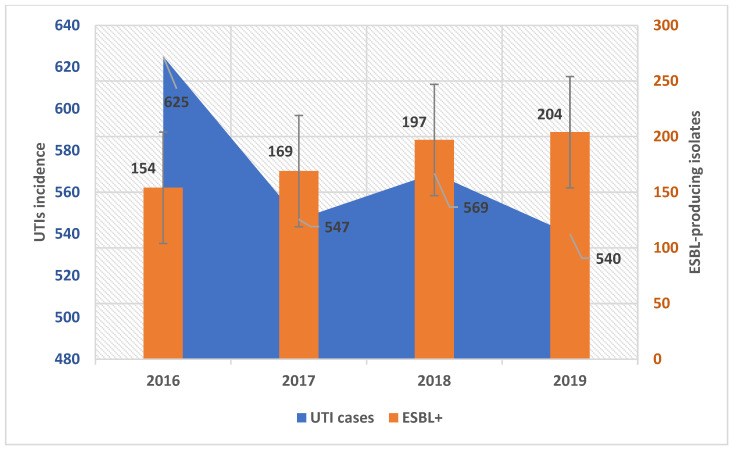
All UTI incidence versus UTIs caused by an ESBL-producing isolate.

During the analyzed timespan, there were 2281 UTIs, of which 1959 had a strain of Enterobacterales as an etiological agent. Of these, we identified 724 (36.9%) positive urine cultures with ESBL-producing Enterobacterales. Notably, although we observed a decrease in the total number of UTIs, at the same time, the share of ESBL-producing isolates steadily increased (Figure 1).

**Figure 2:**
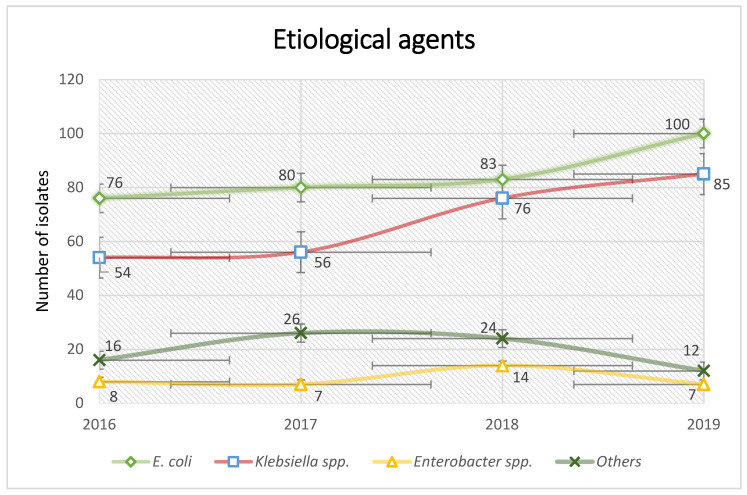
Incidence of ESBL-producing microorganisms during 2016–2019.

The most commonly identified agent was *E. coli*, with a total of 339 strains, followed by *Klebsiella* spp., with 271 strains, and *Enterobacter* spp., with 36 strains; other less prevalent microorganisms were *Proteus* spp.—34 strains, *Providencia* spp.—27 strains, *Serratia* spp.—11 strains, and *M. morganii*—6 strains (Figure 2).

**Figure 3:**
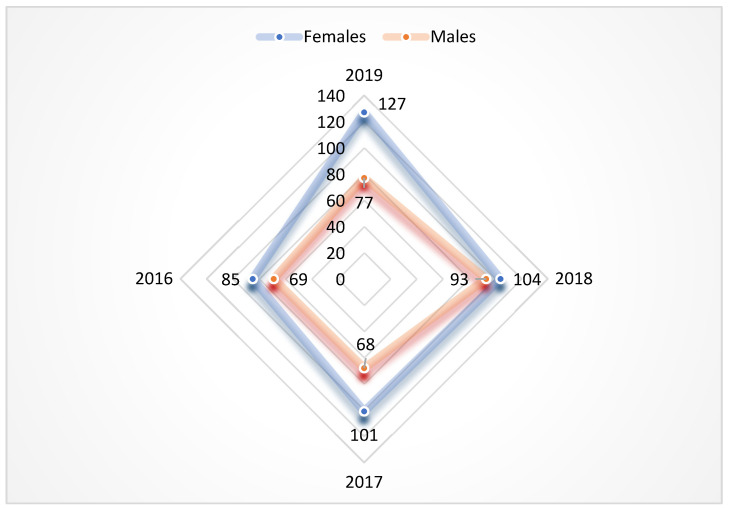
Distribution of patients by gender, 2016–2019.

We observed an association between patients’ age and the presence of an ESBL-producing strain, with 83.3% of the total number of patients aged over 50 years, while almost half (44.8%) of them were over 70 years old, with no significant year-to-year variations. We identified an overall predominance of female patients, accounting for 417 participants (57.6%), compared to 307 (42.4%) men. The largest difference in this regard was recorded in 2019, when out of the total 204 patients, 127 (62.5%) were females (*p* < 0.0001, OR = 2.7204, CI = 1.8229–4.0597) (Figure 3).

We identified 138 (19%) urinary catheterized patients, with the majority of cases occurring in 2019 (42 cases—20.5%) and 2018 (43 cases—21.8%), with only 30 (17.7%) cases in 2017 and 23 (14.9%) in 2016. The presence of an indwelling urinary catheter was correlated with patients’ age (Table 1); moreover, we observed a significant difference between the mean age of urinary catheterized patients compared to non-catheterized patients (68.9 ± 13.7 years vs. 64.2 ± 18.1 years, *p* = 0.026).

**Figure 4:**
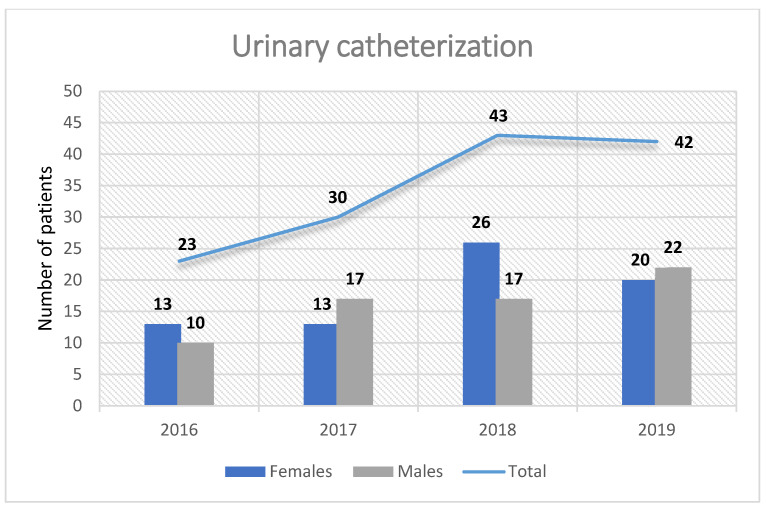
Distribution of urinary catheterized patients by gender.

We observed an increasing trend concerning UTIs associated with urinary catheterization in both men and women, starting from 23 (14.9%) cases in 2016 and subsequently increasing to 42 (20.5%) cases in 2019 (Figure 4).

**Figure 5:**
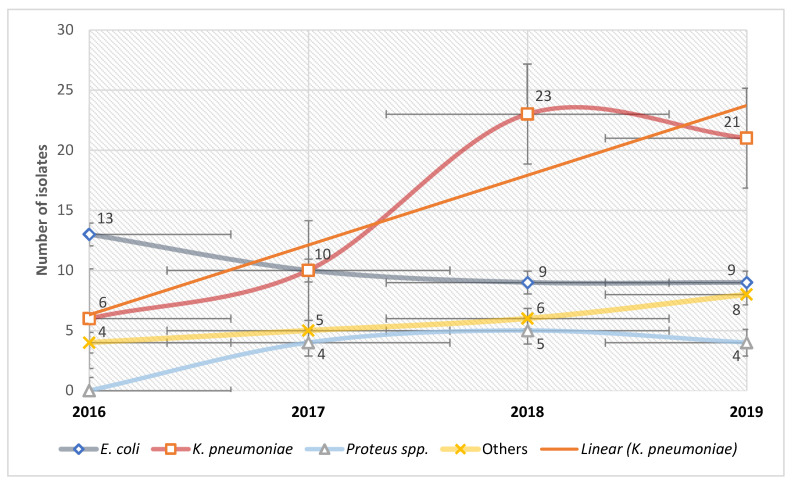
Microorganisms associated with urinary catheterization.

We noted significant shifts in the etiology of UTIs among catheterized patients. While *E. coli* and *K. pneumoniae* remained the predominant pathogens responsible for these infections, we observed a reversal in their ranking over time. Specifically, in 2016, *E. coli* was the primary etiological agent, with a ratio of *E. coli* to *K. pneumoniae* exceeding 2:1. However, by 2019, *K. pneumoniae* emerged as the most frequent etiology, occurring twice as often as *E. coli* (Figure 5).

**Figure 6:**
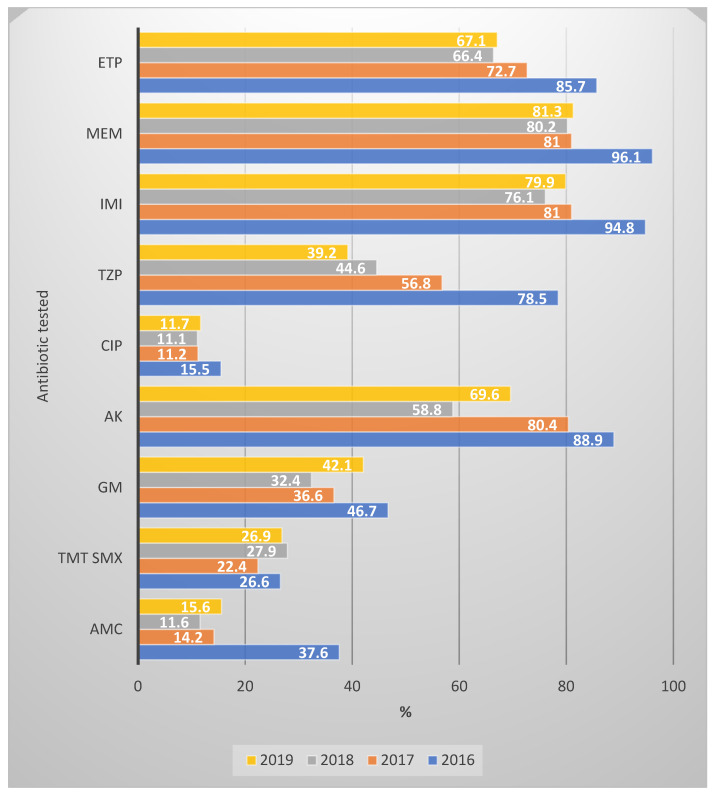
Antibiotic susceptibility rates of ESBL-producing Enterobacterales strains, 2016–2019. AMC—amoxicillin–clavulanic acid; TMT SMX—trimethoprim–sulfamethoxazole; GM—gentamicin; AK—amikacin; CIP—ciprofloxacin; TZP—piperacillin–tazobactam; IMI—imipenem; MEM—meropenem; ETP—ertapenem.

In Figure 6, we can see the evolution of antibiotic susceptibility over the period 2016–2019; basically, with one exception—trimethoprim–sulfamethoxazole susceptibility, which remained relatively constant—we observed a significant decrease in susceptibility to all the tested antibiotics.

Susceptibility to nitrofurantoin and fosfomycin was routinely determined only for *E. coli* strains since EUCAST provides breakpoints for the diameter of inhibition zones only for this microorganism. We identified high fosfomycin susceptibility rates, above 97%, with only four strains resistant to this antibiotic (two in 2017 and two in 2019); in contrast, we found a significant decrease (*p =* 0.0003, OR = 9.8354, CI = 2.2285–43.4092) in susceptibility to nitrofurantoin, from 97.3% in 2016 to 79% in 2019 (Table 2).

Urinary catheterized patients had significantly higher rates of isolated PDR/XDR strains (*p* < 0.0001), in contrast with MDR/UDR isolates, which were negatively correlated with the presence of a urinary catheter; in addition, we observed an important negative correlation between male gender and the UDR pattern, thus mirroring the result that women were more likely to have an infection with a UDR isolate (Table 3).

When assessing the same parameters in correlation with the isolated microorganism, we found that catheterized patients were more likely to have an infection with *Proteus* spp. or *Providencia stuartii*, while *E. coli* had a significant negative correlation with catheterization, male gender, and older age. The only microorganism with significant positive correlations with these parameters was *Klebsiella* spp. (Table 4).

Moreover, *Klebsiella* spp. and *Providencia stuartii* had significant correlations with the PDR pattern, while *E. coli* was more likely to be MDR or even UDR (Table 5).

Further, we aimed to perform a comparative analysis of the susceptibility profile of the identified bacteria, taking into account the presence of a urinary catheter.

Among those patients who did not have a urinary catheter at the time of urine sample collection, we observed a preserved sensitivity to carbapenems of over 85%, with the exception of ertapenem, for which we identified a sensitivity of 74.9%. Sensitivity to the other beta-lactams was low, except for the combination of piperacillin and tazobactam (50.5%), with the highest sensitivity rates found among *E. coli* strains. In addition, a considerable number of strains were sensitive to aminoglycosides, especially amikacin (73.3%, with the highest sensitivity in *Proteus* spp. and *E. coli* strains) (Table 6).

Analyzing the susceptibility profile of urinary catheterized patients, we identified lower susceptibility rates, especially to carbapenems, with less than half of the *Klebsiella* spp. isolates susceptible to these antimicrobials (Table 7 and Table 8).

**Figure 7:**
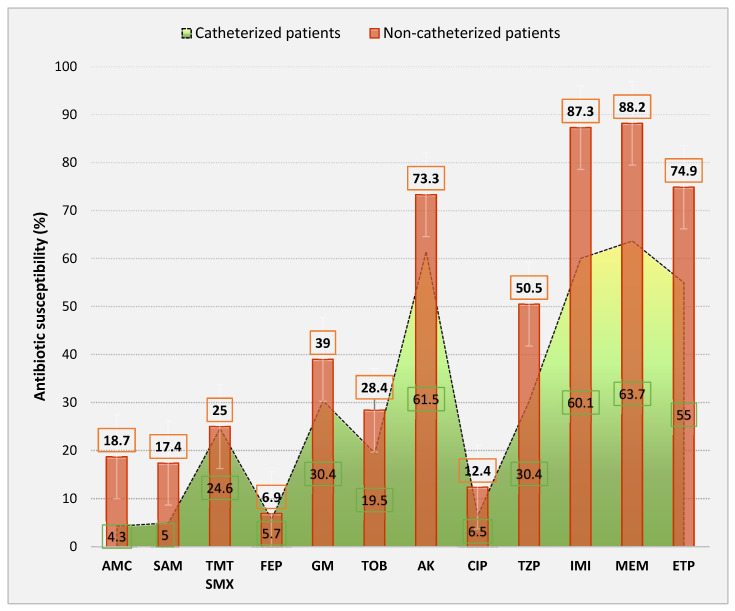
Antibiotic susceptibility (%) by the presence of a urinary catheter. AMC—amoxicillin–clavulanic acid; SAM—ampicillin–sulbactam; TMT SMX—trimethoprim–sulfamethoxazole; FEP—cefepime; GM—gentamicin; TOB—tobramycin; AK—amikacin; CIP—ciprofloxacin; TZP—piperacillin–tazobactam; IMI—imipenem; MEM—meropenem; ETP—ertapenem.

In Figure 7, we can straightforwardly observe the variations in the antibiotic susceptibility rates of isolates from urinary catheterized vs. non-catheterized patients. It can be ascertained that, for all tested antibiotics, susceptibility was significantly lower among catheterized patients, with the most important differences recorded in the case of carbapenems regardless of whether we refer to imipenem (60.1% catheterized patients vs. 87.3% non-catheterized patients, *p* < 0.00001, OR = 0.2181, CI = 0.1435–0.3316), meropenem (63.7% vs. 88.2%, *p* < 0.00001, OR = 0.2349, CI = 0.1530–0.3605), or ertapenem (55% vs. 74.9%, *p* < 0.00001, OR = 0.4105, CI = 0.2796–0.6026), but also for piperacillin–tazobactam (30.4% vs. 50.5%, *p* = 0.00002, OR = 0.4286, CI = 0.2881–0.6376), ciprofloxacin (6.5% vs. 12.4%, *p* = 0.0477, OR = 0.4903, CI = 0.2389–1.0061), amikacin (61.5% vs. 73.3%, *p* = 0.0059, OR = 0.5818, CI = 0.3944–0.8584), gentamicin (30.4% vs. 39%, *p* = 0.05, OR = 0.6820, CI = 0.4577–1.0162) as well as for combinations with beta-lactamase inhibitors, such as ampicillin + sulbactam (5% vs. 17.4%, *p* = 0.0002, OR = 0.2536, CI = 0.1151–0.5585) or amoxicillin + clavulanic acid (4.3% vs. 18.7%, *p* = 0.00003, OR = 0.1957, CI = 0.0846–0.4574).

## 3. Discussion

ESBL-producing Enterobacterales have become a significant clinical and epidemiological concern. These strains were first discovered in the early 1980s and since then have ubiquitously spread, becoming endemic and related to both community-acquired and hospital-associated infections. They are accompanied by increased medical costs and adverse patient outcomes such as prolonged length of hospitalization, therapeutic failure, and increased mortality. In 2017, more than 197,000 cases of ESBL-producing microorganism infections were reported in the United States alone [12]. Broad-spectrum antibiotics, such as carbapenems, are usually required for the treatment of these infections, but carbapenem resistance is also on the rise [13,14,15], an alarming situation also confirmed by our findings.

The significant per capita antibiotic consumption in Romania, coupled with ineffective measures in terms of intra-hospital infection prophylaxis, is the basis for the increased rates of AMR in this geographical area. Both national and international multicenter studies support this hypothesis, mentioning that for some antimicrobials, epidemiological alerts have even been issued due to their alarming resistance rate [16,17,18,19,20].

Although UTIs in catheterized patients usually have lower morbidity and mortality rates than other healthcare-associated infections (HAIs), the use of catheters is still a significant burden on healthcare systems. Inappropriate use of catheters is rampant, with estimates suggesting that 15% to 25% of hospitalized patients receive short-term urinary catheterization [21,22,23,24]. Furthermore, every day a patient retains an indwelling catheter, their risk of developing a CAUTI increases by 3–7% [25,26,27].

CAUTIs represent the most common device-accompanying HAIs, along with ventilator-associated pneumonia or central-line-associated bloodstream infections [28]. Most of the recent studies focusing on nosocomial infections point out that the incidence of CAUTIs caused by Enterobacterales, particularly *Klebsiella* spp., has significantly increased [29,30].

An ample study that analyzed UTI incidence from 1990 to 2019, including data from 203 countries and territories, also showed a significant increase in UTI incidence, by 60.4%. In addition, they also reported a significant increase in the severity of this pathology, manifested in an increased UTI-attributable mortality rate by 140.1% but also an increase in disability-adjusted life-years by 68.8% [31].

Antibiotic resistance poses a significant risk, potentially leading to the selection of inappropriate therapy and the recurrence of infections, which is a common issue among patients with UTIs [32,33]. Specifically, patients infected with ESBL-producing Gram-negative bacilli experience over twice as many hospital readmissions within 30 days of their initial discharge compared to those infected with non-ESBL-producing Enterobacterales (26.8% versus 12.4%) [34]. Moreover, the inadequate empirical treatment of UTIs may heighten the likelihood of systemic infections, resulting in substantially increased morbidity and mortality rates [35,36]. For instance, in a study focusing on sepsis cases caused by ceftriaxone-resistant *E. coli* or *K. pneumoniae*, the urinary tract was identified as the primary source of infection in the majority of cases, accounting for 60.9% [37].

Also analyzing the evolution of the incidence of ESBL-producing Enterobacterales strains on a pool of 876,507 isolates, Aronin et al. identified a significant increase in microorganisms with an ESBL phenotype, from 8.9% in 2011 to 14.2% in 2020, with an associated increase in ESBL-producing resistance rates per 1000 admissions from 3.7 in 2011 to 6.4 in 2020 [38]. This is consistent with our findings, as we found that the percentage of ESBL-producing *E. coli* increased significantly between 2016 and 2019, from 19% to 27.8% (*p* = 0.004). In their analysis of 876,507 isolates, Aronin et al. observed a noteworthy rise in the incidence of ESBL-producing Enterobacterales strains over time. Specifically, they noted a substantial increase in microorganisms exhibiting an ESBL phenotype, from 8.9% in 2011 to 14.2% in 2020 [38]. These findings align with our own research, where we observed a significant increase in the percentage of ESBL-producing *E. coli* from 19% to 27.8% between 2016 and 2019 (*p* = 0.004).

The majority of strains were isolated from women, representing 57.6% of the total number of tested patients, a figure that has even reached a peak of 62.5% in 2019. This aspect is supported by the fact that the female gender, due to its associated anatomical peculiarities, is considered an additional, non-modifiable risk factor for the development of UTI, regardless of the etiological agent but especially among urinary catheterized patients [39,40].

Our analysis of CAUTI etiology revealed notable variations depending on the year under examination. While *E. coli* and *K. pneumoniae* remained the primary culprits, significant shifts were observed. Initially, *E. coli* predominated in 2016; however, from 2018 onwards, *K. pneumoniae* emerged as the predominant etiological agent, supplanting *E. coli* in this role. This shift highlights dynamic changes in the microbial landscape of CAUTIs and underscores the importance of ongoing surveillance and the adaptation of treatment strategies. Most studies in the literature classify *E. coli* as the main etiologic agent of CAUTIs [40,41,42], although we also identified studies reporting *K. pneumoniae* as the most common Enterobacterales isolate [43,44]. In certain European countries, the incidence of CAUTIs attributed to ESBL-producing *E. coli* currently stands at less than 5% [32]. However, in countries like Spain, this figure can escalate to as high as 23.6%, while in Turkey, it reaches an even more alarming rate of 38.2% [45].

It is also worth mentioning that among urinary catheterized patients, it has been demonstrated that microorganisms often persist, despite catheter replacements or antimicrobial therapy, because of the formation of bacterial biofilms on the catheter and bacterial communities within bladder epithelial cells [46]. Among Enterobacterales, the most important pathogen involved in biofilm formation is *Proteus* spp. [46], positively correlated in our study with urinary catheterization (*p* = 0.02). Moreover, biofilms aid in the exchange and recycling of nucleic acids because cells remain in close proximity within the extracellular polymeric substances matrix for extended periods, serving to protect microorganisms in challenging environments [47]. Notably, genetic material is transmitted through both vertical and horizontal gene transfer within biofilms. This mechanism has the potential to convert innocuous bacteria into significant human pathogens [48].

A study by Albaramki et al. investigating the incidence and impact of UTIs caused by ESBL-producing Enterobacterales revealed elevated rates of resistance to various antibiotics. Specifically, resistance rates were notably high for amoxicillin + clavulanic acid (94.8%), third-generation cephalosporins (98.7%), fluoroquinolones (54.5%), and gentamicin (54.5%). However, resistance to amikacin (32.5%), and particularly, carbapenems (1.3%) remained significantly lower [49]. Similarly, Vachvanichsanong et al. reported increased resistance percentages to cephalosporins (99–100%) and aminopenicillins (99%), but with much lower resistance rates to fluoroquinolones (44%), piperacillin + tazobactam (22%), or amikacin (11%). Notably, none of the isolates were found to be resistant to carbapenems in their study [50], as well as in Ziółkowski et al.’s study [51].

A recent study, conducted in China, analyzing the antibiotic susceptibility profile of strains of *E. coli* strains isolated from patients with community-acquired UTIs showed increased resistance rates to common cephalosporins (93.4% resistance to cefotaxime, 93.1% to ceftriaxone, and 76.8% to cefepime), while only 8.1% resistance to the combination of cefoperazone and sulbactam was observed. In addition, they reported very low resistance to amikacin (3%), nitrofurantoin (2.7%), fosfomycin (8.4%), or carbapenems (0.3% resistance to imipenem and 0.6% to ertapenem) [45]. As in our study, resistance to fluoroquinolones (72.3%) or trimethoprim–sulfamethoxazole (68.7%) was identified in increased percentages [45].

In the analysis by Aronin et al., although they identified high resistance rates to the antibiotics tested (64.6% to beta-lactams, 29.3% to fluoroquinolones, 26.3% to trimethoprim–sulfamethoxazole, and 27.6% to nitrofurantoin), they found a lower percentage of resistant strains [38] over the period 2011–2022, in contrast to our study, in which we recorded a decreased susceptibility rate for almost all the tested antibiotics.

With the antibiotic drug development pipeline struggling to keep pace with rising resistance rates, prevention and a deeper understanding of CAUTIs are fundamental in addressing this challenge. By focusing on preventive measures such as minimizing catheter use, employing alternative catheterization methods, implementing strict infection control practices, and promoting judicious antibiotic use, healthcare providers can significantly reduce the incidence of CAUTIs [52]. Additionally, continued research into the pathogenesis of CAUTIs and the mechanisms underlying antimicrobial resistance can inform targeted interventions and improve patient outcomes [53]. There are several interventions aimed at reducing the occurrence of CAUTIs. Among these, the duration of catheterization emerges as the most crucial factor; therefore, the primary intervention to mitigate CAUTI risk is minimizing the use of indwelling catheters and promptly removing them when medically appropriate [54]. External catheters represent a viable alternative to indwelling catheters and are recommended by the Centers for Disease Control and Prevention [55].

Our study is burdened by certain limitations. For example, the study population is limited to a single region in Romania, and the findings may not reflect antibiotic resistance patterns in other regions of the country. It should be mentioned that we are currently working on a prospective multicenter analysis in which we study not only BLSE production but also the prevalence and risk factors for carbapenemase production. Yet, another notable limitation is the lack of genetic studies or sequencing of the isolated strains. Additional sequencing and subsequent analyses would have offered a more comprehensive insight into the epidemiology of CAUTIs in our region. Having incomplete patient data files was another limitation, which is related to the retrospective design of the study.

## 4. Materials and Methods

In our retrospective study, our objective was to conduct a comprehensive analysis of the incidence of ESBL-producing Enterobacterales in urine samples collected between 1 January 2016 and 31 December 2019 at the “St. Parascheva” Clinical Hospital of Infectious Diseases in Iasi. This hospital is a 300-bed university setting and serves as the largest tertiary center for infectious diseases in northeastern Romania. This region has an approximate population of 4 million inhabitants.

Study population: Our study included all consecutive, non-duplicate ESBL-producing Enterobacterales strains isolated from urine cultures with a bacterial count of ≥10^5^ colony-forming units per milliliter (CFU/mL). These criteria ensured the inclusion of clinically significant infections while minimizing the inclusion of contaminant samples.

Antibiotic susceptibility testing was performed by the Kirby Bauer diffusimetric method using EUCAST tables with breakpoints in effect at the time of strain isolation (v6.0 for 2016, v7.0 and v7.1 for 2017, v8.0 and v8.1 for 2018, and v9.0 for 2019) [56] for the interpretation of MICs and inhibition zone diameters. The following antibiotic discs were used: ampicillin (10 µg); ampicillin–sulbactam (10–10 µg); amoxicillin–clavulanic acid (20–10 µg); piperacillin–tazobactam (30–6 µg); cefepime (30 µg); cefixime (5 µg); cefotaxim (5 µg); cefoxitin (30 µg); ceftazidime (10 µg); ceftazidime–avibactam (10–4 µg); cefuroxime (30 µg); ertapenem (10 µg); imipenem (10 µg); meropenem (10 µg); aztreonam (30 µg); ciprofloxacin (5 µg); levofloxacin (5 µg); moxifloxacin (5 µg);) norfloxacin (10 µg); ofloxacin (5 µg); amikacin (30 µg); gentamicin (10 µg); tobramycin (10 µg); fosfomycin (200 µg)—only for *E. coli*; nitrofurantoin (100 µg)—only for *E. coli*; trimethoprim–sulfamethoxazole (1.25–23.75 µg).

Isolates were classified as follows [57]:

- MDR—isolates non-susceptible to at least one agent from ≥3 antimicrobial classes;
- XDR—isolates non-susceptible to at least one antibiotic from all but a maximum of two antimicrobial classes;
- PDR—isolates non-susceptible to all antibiotics from all classes;
- UDR—isolates not fully susceptible (wild types) but easily treated with standard therapies [58].

Furthermore, we conducted a comparative analysis of patient characteristics based on the presence of an indwelling urinary catheter, examining demographics, infection etiology, and the susceptibility profile of isolated bacteria.

Data collection: To collect data for our study, we accessed records from the Medical Analysis Laboratory of the Infectious Diseases Hospital “Sf. Parascheva” in Iasi. These records provided information on urine cultures performed during the study period. In conjunction with statistical data, including diagnoses at discharge, we were able to identify cases that met the criteria for UTIs, thereby excluding cases of asymptomatic bacteriuria or colonization. These records allowed us to extract information on various demographic characteristics of the patients, including age and gender, as well as the presence of a permanent urinary catheter. Additionally, we obtained data on the etiological agent responsible for the infection and the antibiotic susceptibility profile, specifically identifying ESBL-producing strains. This comprehensive dataset enabled us to explore the potential correlations between catheterization status and infection characteristics, aiding in a deeper understanding of UTIs in this patient population.

Statistical analysis: Categorical variables are presented as numbers and percentages, with continuous variables being presented as means and standard deviations. We used the 95% confidence interval in parameter estimation. Independent *t*-tests were used to compare continuous variables, while chi-squared tests were used to compare categorical variables. A *p*-value < 0.05 was considered statistically significant. For initial data collection, we used Microsoft Excel 2016 version (Microsoft Corporation, Redmond, WA, USA), while the data analysis was performed with SPSS version 23 (IBM, Armonk, VA, USA).

## 5. Conclusions

Our study reflected the multifaceted clinical, microbiological, or epidemiological aspects related to the dynamic trend of CAUTIs, shedding light on the challenges related to multidrug resistance. Not only was the urinary catheterization per se correlated with significant antibiotic resistance, but we also outlined a worrisome temporal trend, with a decreasing susceptibility between 2016 and 2019. It is imperative to recognize the increasing concern of antibiotic resistance as it relates to CAUTI management. Therefore, appropriate measures should be taken to curb the inappropriate use of catheters and ensure that empiric therapy is accurate, thereby reducing the risk of recurrent infections and systemic infections, ultimately leading to improved patient outcomes.

There is a need for further advancement in the development of intraurethral options as substitutes for indwelling catheterization in both men and women. Evaluations to determine whether these devices effectively decrease the risk of CAUTIs are essential. Significant progress in preventing these infections will necessitate the development of biomaterials capable of preventing or restricting biofilm formation. Ultimately, a comprehensive approach that prioritizes prevention and a detailed understanding of CAUTIs are essential aspects in combating this increasingly prevalent public health issue.

## Figures and Tables

**Table 1:** Correlations between urinary catheterization and patient’s age and gender.

			Age	Male Gender
Spearman’s rho	Urinary catheterization	Correlation Coefficient	0.083 *	0.053
		Sig. (2-tailed)	0.026	0.152

* Spearman correlation is significant at the 0.05 level (2-tailed). Green—positive correlation

**Table 2:** *E. coli*’s susceptibility to nitrofurantoin and fosfomycin.

	Nitrofurantoin N, (%)	Fosfomycin N, (%)
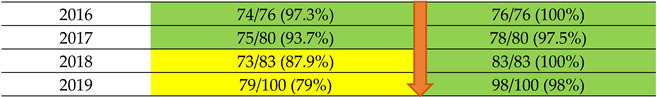		
*p*-value	0.0003	0.601

Yellow—susceptibility between 70.1% and 90% (antibiotics that can be prescribed empirically in mild to moderate infection); Green—susceptibility > 90% (antibiotics that can be selected as empirical therapy even in severe infections).

**Table 3:** Correlations between antibiotic susceptibility patterns and demographic aspects or the presence of a urinary catheter.

			Age	Urinary Catheterization	Male Gender
Spearman’s rho	PDR	Correlation Coefficient	0.022	0.193 **	−0.003
		Sig. (2-tailed)	0.559	<0.0001	0.927
	XDR	Correlation Coefficient	0.037	0.145 **	0.080 *
		Sig. (2-tailed)	0.318	<0.0001	0.030
	MDR	Correlation Coefficient	−0.011	−0.206 **	−0.015
		Sig. (2-tailed)	0.758	<0.0001	0.692
	UDR	Correlation Coefficient	−0.043	−0.069	−0.106 **
		Sig. (2-tailed)	0.248	0.063	0.004

** Spearman correlation is significant at the 0.01 level (2-tailed). * Spearman correlation is significant at the 0.05 level (2-tailed). Green—positive correlation; Red—negative correlation. MDR—multidrug resistance, XDR—extensive drug resistance, PDR—pandrug resistance, UDR—usual drug resistance.

**Table 4:** Correlations between the isolated microorganism and the demographic aspects of urinary catheterization.

		**Male Gender**	**Urinary Catheterization**	**Age**
*E. coli*	Pearson Correlation	−0.139 **	−0.159 **	−0.117 **
	Sig. (2-tailed)	<0.0001	<0.0001	0.002
*Klebsiella* spp.	Pearson Correlation	0.086 *	0.056	0.143 **
	Sig. (2-tailed)	0.020	0.131	<0.0001
*Enterobacter* spp.	Correlation Coefficient	−0.013	−0.039	−0.039
	Sig. (2-tailed)	0.721	0.300	0.297
*Proteus* spp.	Correlation Coefficient	0.092 *	0.087 *	0.015
	Sig. (2-tailed)	0.013	0.020	0.696
*Morganella morganii*	Correlation Coefficient	0.045	0.072	0.052
	Sig. (2-tailed)	0.228	0.053	0.159
*Serratia marcescens*	Correlation Coefficient	0.053	−0.003	−0.056
	Sig. (2-tailed)	0.151	0.940	0.133
*Providencia stuartii*	Correlation Coefficient	−0.007	0.183 **	−0.057
	Sig. (2-tailed)	0.859	<0.0001	0.125

** Spearman correlation is significant at the 0.01 level (2-tailed). * Spearman correlation is significant at the 0.05 level (2-tailed). Green—positive correlation; Red—negative correlation.

**Table 5:** Correlations between the resistance pattern and the isolated microorganism.

Correlations					
		PDR	XDR	MDR	UDR
*Providencia stuartii*	Correlation Coefficient	0.466 **	0.031	−0.280 **	−0.047
	Sig. (2-tailed)	<0.0001	0.398	<0.0001	0.207
*E. coli*	Correlation Coefficient	−0.269 **	−0.332 **	0.359 **	0.119 **
	Sig. (2-tailed)	<0.0001	<0.0001	<0.0001	0.001
*Proteus* spp.	Correlation Coefficient	−0.068	0.016	0.045	−0.030
	Sig. (2-tailed)	0.066	0.667	0.225	0.423
*Klebsiella* spp.	Correlation Coefficient	0.136 **	0.277 **	−0.257 **	−0.082 *
	Sig. (2-tailed)	<0.0001	<0.0001	<0.0001	0.027
*Serratia marcescens*	Correlation Coefficient	0.007	−0.015	0.023	−0.030
	Sig. (2-tailed)	0.851	0.690	0.539	0.426
*Enterobacter* spp.	Correlation Coefficient	−0.038	0.111 **	−0.065	0.007
	Sig. (2-tailed)	0.312	0.003	0.080	0.861
*Morganella morganii*	Correlation Coefficient	0.031	0.010	−0.015	−0.022
	Sig. (2-tailed)	0.400	0.797	0.694	0.558

** Spearman correlation is significant at the 0.01 level (2-tailed). * Spearman correlation is significant at the 0.05 level (2-tailed). Green—positive correlation; Red—negative correlation. MDR—multidrug resistance, XDR—extensive drug resistance, PDR—pandrug resistance, UDR—usual drug resistance.

**Table 6:** Antibiotic susceptibility for strains isolated from non-catheterized patients.

Antibiotic Tested	*E. coli*	*Enterobacter* spp.	*Klebsiella* spp.	*Proteus* spp.	*Providencia* spp.	*M. morganii*	*S. marcescens*	Total
AMC	26.5%	IR	13.3%	14.2%	IR	IR	IR	18.7%
SAM	28.9%	IR	6.1%	14.2%	IR	IR	IR	17.4%
TMT-SMX	33.3%	14.7%	19%	4.7%	0	0	22.2%	25%
CXM	1%	2.9%	0.4%	4.7%	0	0	IR	1%
CAZ	7.7%	2.9%	1.9%	4.7%	0	0	0	4.9%
FEP	5.7%	11.7%	3.8%	52.3%	9%	0	0	6.9%
GM	47.8%	26.4%	28.5%	52.3%	9%	33.3%	55.5%	39%
TOB	38%	14.7%	20.4%	14.2%	18.1%	0	11.1%	28.4%
AK	84.8%	58.8%	60.9%	95.2%	18.1%	33.3%	77.7%	73.3%
CIP	13.1%	20.5%	11.9%	4.7%	0	0	11.1%	12.4%
TZP	60.2%	38.2%	37.1%	85.7%	27.2%	66.6%	33.3%	50.5%
IMI	99.6%	76.4%	78.5%	66.6%	18.1%	66.6%	77.7%	87.3%
MEM	100%	76.4%	77.6%	95.2%	18.1%	66.6%	77.7%	88.2%
ETP	90.5%	47%	60.4%	70.8%	18.1%	66.6%	66.6%	74.9%

AMC—amoxicillin–clavulanic acid; SAM—ampicillin–sulbactam; TMT-SMX—trimethoprim–sulfamethoxazole; CXM—cefixime; CAZ—ceftazidime; FEP—cefepime; GM—gentamicin; TOB—tobramycin; AK—amikacin; CIP—ciprofloxacin; TZP—piperacillin–tazobactam; IMI—imipenem; MEM—meropenem; ETP—ertapenem; IR—intrinsic resistance. Red—susceptibility < 50% (antibiotics firmly not recommended as empirical therapy); Orange—susceptibility between 50% and 70% (antibiotics that should be avoided as empirical therapy); Yellow—susceptibility between 70.1% and 90% (antibiotics that can be prescribed empirically in mild to moderate infection); Green—susceptibility > 90% (antibiotics that can be selected as empirical therapy even in severe infections); Gray—N/A.

**Table 7:** Antibiotic susceptibility for strains isolated from urinary catheterized patients.

Antibiotic Tested	*E. coli*	*Enterobacter* spp.	*Klebsiella* spp.	*Proteus* spp.	*Providencia* spp.	*M. morganii*	*S. marcescens*	Total
AMC	28.5%	IR	5%	7.6%	IR	IR	IR	4.3%
SAM	11.9%	IR	1.6%	7.6%	IR	IR	IR	5%
TMT-SMX	38%	25%	22%	30.7%	6.6%	33.3%	0	24.6%
CXM	0	0	0	0	0	0	IR	0
CAZ	7.1%	0	1.6%	7.6%	0	33.3%	0	4.3%
FEP	2.3%	0	1.6%	38.4%	0	33.3%	0	5.7%
GM	52.3%	0	18.6%	53.8%	0	33.3%	50%	30.4%
TOB	38%	0	11.8%	30.7%	0	0	0	19.5%
AK	90.4%	50%	50.8%	76.9%	6.6%	100%	50%	61.5%
CIP	4.7%	25%	5%	15.3%	0	33.3%	0	6.5%
TZP	66.6%	25%	13.5%	92.3%	6.6%	33.3%	50%	30.4%
IMI	100%	100%	44%	76.9%	0	0	50%	60.1%
MEM	100%	100%	42.3%	100%	0	66.6%	100%	63.7%
ETP	92.8%	75%	32.2%	100%	0	33.3%	50%	55%

AMC—amoxicillin–clavulanic acid; SAM—ampicillin–sulbactam; TMT-SMX—trimethoprim–sulfamethoxazole; CXM—cefixime; CAZ—ceftazidime; FEP—cefepime; GM—gentamicin; TOB—tobramycin; AK—amikacin; CIP—ciprofloxacin; TZP—piperacillin–tazobactam; IMI—imipenem; MEM—meropenem; ETP—ertapenem; IR—intrinsic resistance. Red—susceptibility < 50% (antibiotics not recommended as empirical therapy); Orange—susceptibility between 50% and 70% (antibiotics that should be avoided as empirical therapy); Yellow—susceptibility between 70.1% and 90% (antibiotics that can be prescribed empirically in mild to moderate infection); Green—susceptibility > 90% (antibiotics that can be selected as empirical therapy even in severe infections); Gray—N/A.

**Table 8:** Correlation between urinary catheterization and carbapenem-resistant microorganisms.

			IMI	MEM	ETP
Spearman’s rho	Urinary catheterization	Correlation Coefficient	0.273 **	0.249 **	0.228 **
		Sig. (2-tailed)	<0.0001	<0.0001	<0.0001

** Spearman correlation is significant at the 0.01 level (2-tailed). IMI—imipenem; MEM—meropenem; ETP—ertapenem. Green—positive correlation.

## Data Availability

All the data are found in the manuscript.

## Abbreviation

AK	amikacin
AMR	antimicrobial resistance
AMC	amoxicillin–clavulanic acid
CAUTIs	catheter-associated urinary tract infections
CAZ	ceftazidime
CFU	Colony-forming units
CI	confidence interval
CIP	ciprofloxacin
CXM	cefixime
ESBLs	extended-spectrum beta-lactamases
ETP	ertapenem
FEP	cefepime
GM	gentamicin
HAI	healthcare-associated infection
IMI	imipenem
IR	intrinsic resistance
MDR	multidrug resistance
MEM	meropenem
OR	odds ratio
PDR	pandrug resistance
SAM	ampicillin–sulbactam
TMT-SMX	trimethoprim–sulfamethoxazole
TOB	tobramycin
TZP	piperacillin–tazobactam
UDR	usual drug resistance
UTIs	urinary tract infections
XDR	extensive drug resistance
